# Supplementary material for: TRABID inhibition activates cGAS/STING-mediated anti-tumor immunity through mitosis and autophagy dysregulation
Source: Nat Commun. 2023 May 26;14:3050. doi: 10.1038/s41467-023-38784-z (PMC10220035; doi:10.1038/s41467-023-38784-z)
Supplement: Supplementary file 1 — Supplementary Information [file 41467_2023_38784_MOESM1_ESM.pdf]

Supplementary Information

TRABID inhibition activates cGAS/STING-mediated anti-tumor immunity through mitosis and autophagy dysregulation

Supplementary Figures 1-8

Supplementary Tables 1-3

Supplementary Figures

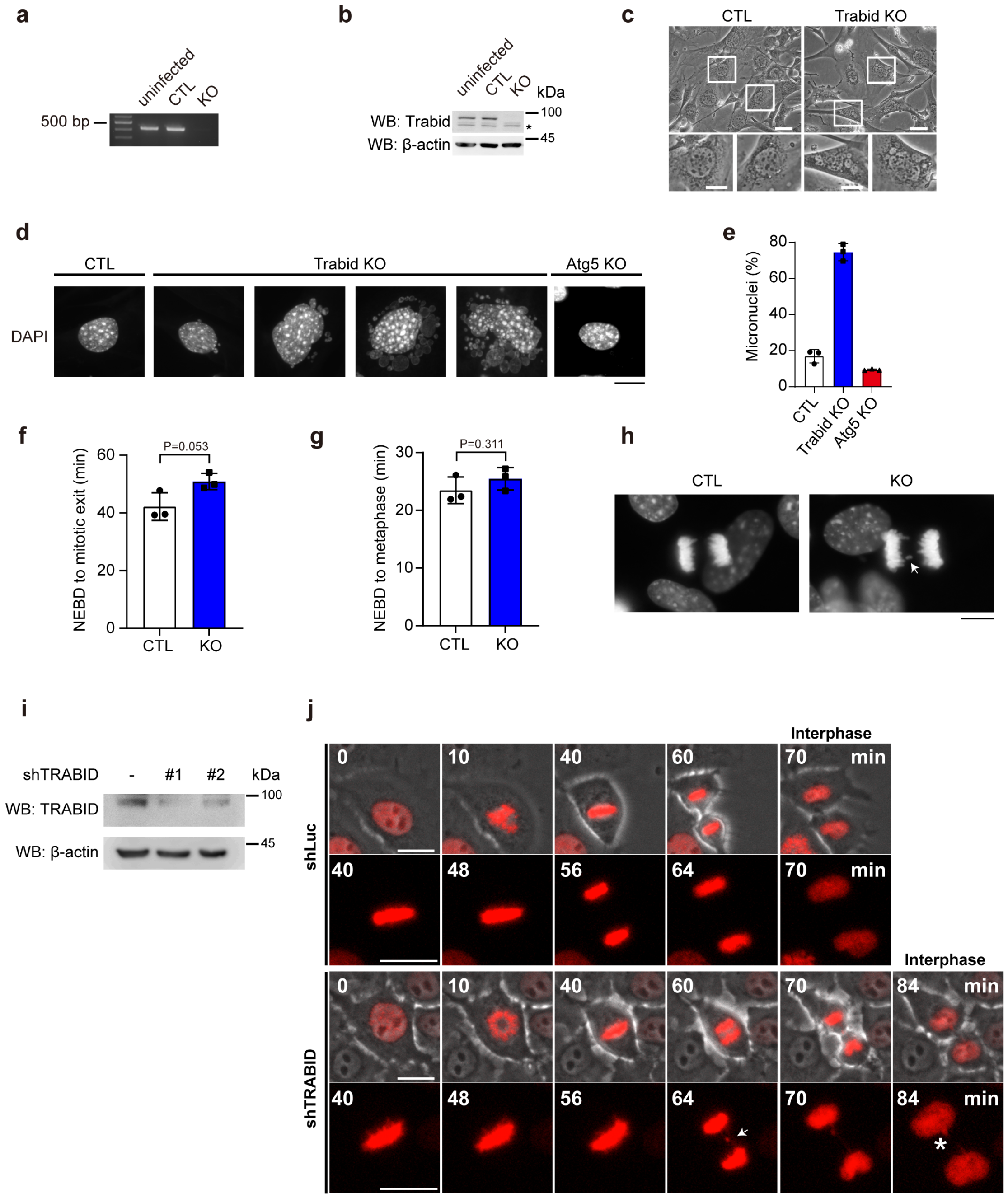

Supplementary Fig. 1. TRABID deficiency induces mitotic errors and micronuclei. (a, b) PCR and Western blot analyses of *Zranb1*$^{flox/flox}$ MEFs infected with AdLuc (CTL) or AdCre (KO). Asterisk denotes a non-specific band. (c) Phase contrast images of Trabid KO and control MEFs. The boxed areas are enlarged to show below for revealing the micronuclei. Bars, 20 µm. (d, e) DAPI staining of

control MEFs or Trabid KO MEFs cultured for 4 days after adenovirus infection or Atg5 KO MEFs. Bar, 20 μm. The percentage of cells showing micronuclei was counted. Data are mean ± SD (n=3 independent experiments and > 200 cells per group per experiment were counted). (f, g) The durations from NEBD to mitotic exit (defined by cell re-attachment) and NEBD to metaphase of indicated cell types were counted. Data are mean ± SD (n=3 independent experiments and >50 cells per group per experiment were counted). Each color represents an individual experiment. P values are determined by two-side Student's t-test. (h) Representing image showing lagging chromosome (arrow) in an anaphase cell. Bar, 10 μm. (i) Western blot analysis of TRABID expression in HeLa cells stably expressing indicated shRNAs and H2B-mCherry. (j) Time-lapse microscopic analysis of the mitotic cell division of HeLa cells stably expressing control or TRABID shRNA and H2B-mCherry. Images were taken at 2 min intervals between acquisitions. Time (min) is given relative to the first time frame in late G2 phase. Lagging chromosome is marked by an arrowhead. Micronuclei in the interphase cells are marked by an asterisk. Bars, 20 μm. For (a, b, h-j), blots or image are representatives of three independent experiments. Source data are provided as a Source Data file.

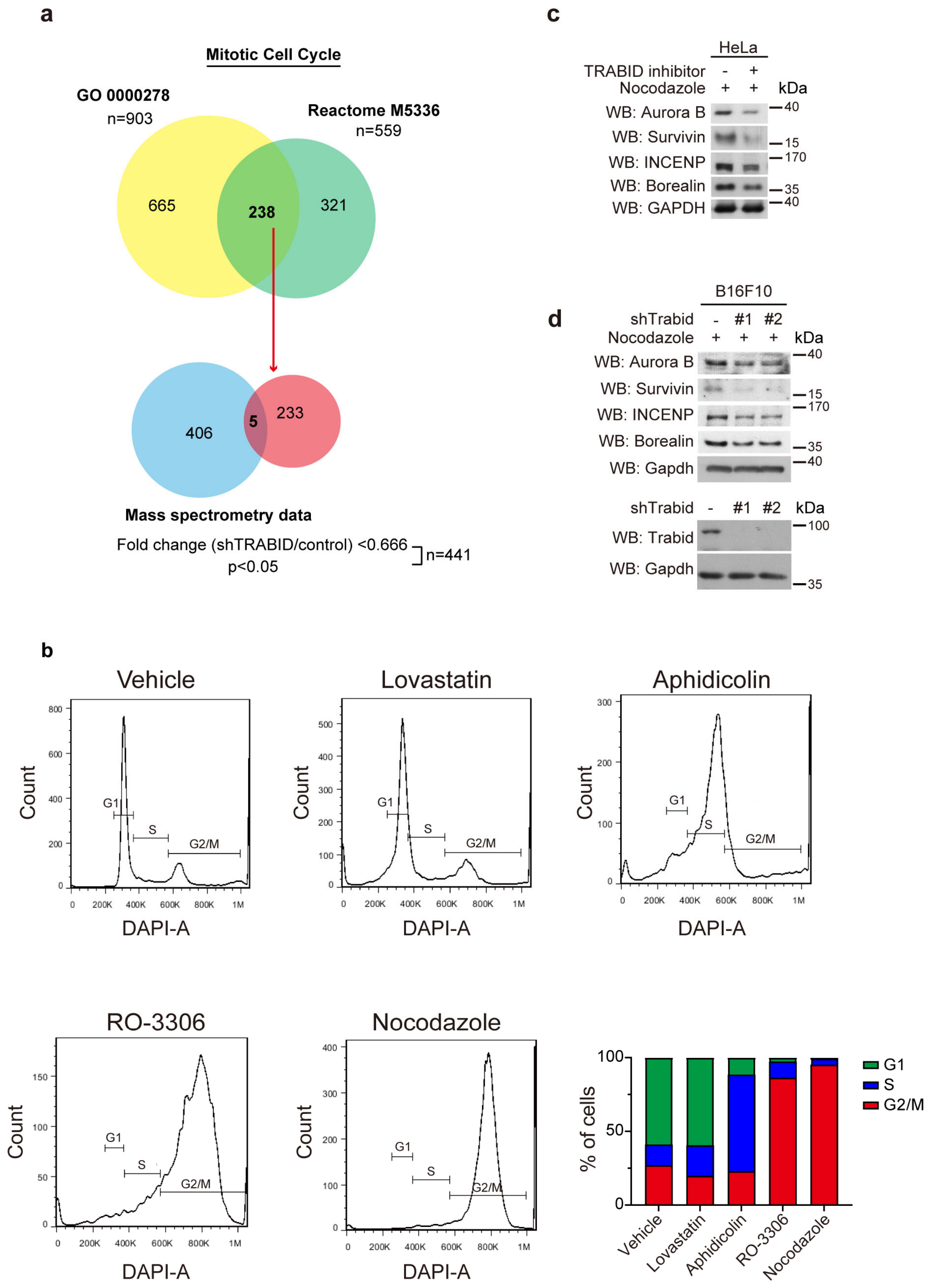

Supplementary Fig. 2. TRABID ablation downregulates CPC. (a) Venn diagram showing the overlap of proteins that are downregulated by TRABID knockdown with that are listed in the “mitotic cell cycle” category of both GO and Reactome pathway analytic tools. P values are determined by two-sided Student’s t-test. (b) Flow

cytometry analysis of cell cycle distribution of HeLa cells treated as in Fig. 2c. (c, d) Western blot analysis of CPC components in HeLa cells treated with 3 μM nocodazole and 3 μM TRABID inhibitor for 18 h (c), or B16F10 cells stably expressing control or Trabid shRNAs and treated with 3 μM nocodazole for 18 h (d). The knockdown efficiencies are shown in d. Blots are representatives of three independent experiments. Source data are provided as a Source Data file.

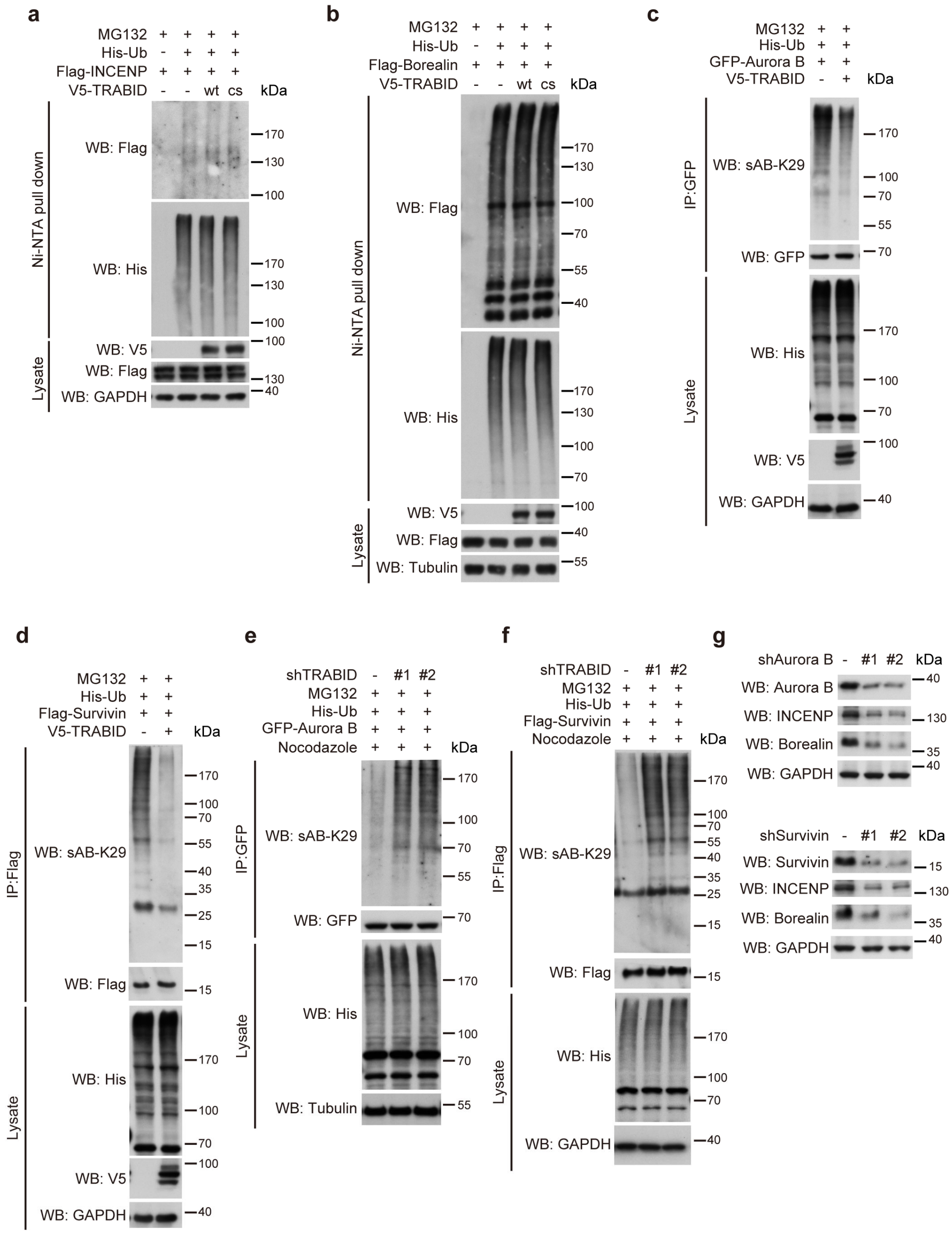

Supplementary Fig. 3. TRABID promotes K29-deubiquitination of Aurora B and Survivin without affecting INCENP and Borealin ubiquitination. (a, b) Western blot analysis of INCENP or Borealin ubiquitination in 293T cells transfected with indicated constructs. Cells were treated with MG132 to preserve the ubiquitination signals. (c-f) Analysis of K29-linked ubiquitination of GFP-Aurora B or Flag-Survivin in 293T cells transfected with indicated constructs (c, d) or HeLa derivatives

as in Fig. 2e transfected with indicated constructs and treated with 3 μM nocodazole for 18 h (e, f). Cells were treated with MG132 to preserve the ubiquitination signals. (g) Western blot analysis of indicated proteins in HeLa cells stably expressing Aurora B shRNAs or Survivin shRNAs. Blots are representatives of three (for a-f) or two (for g) independent experiments. Source data are provided as a Source Data file.

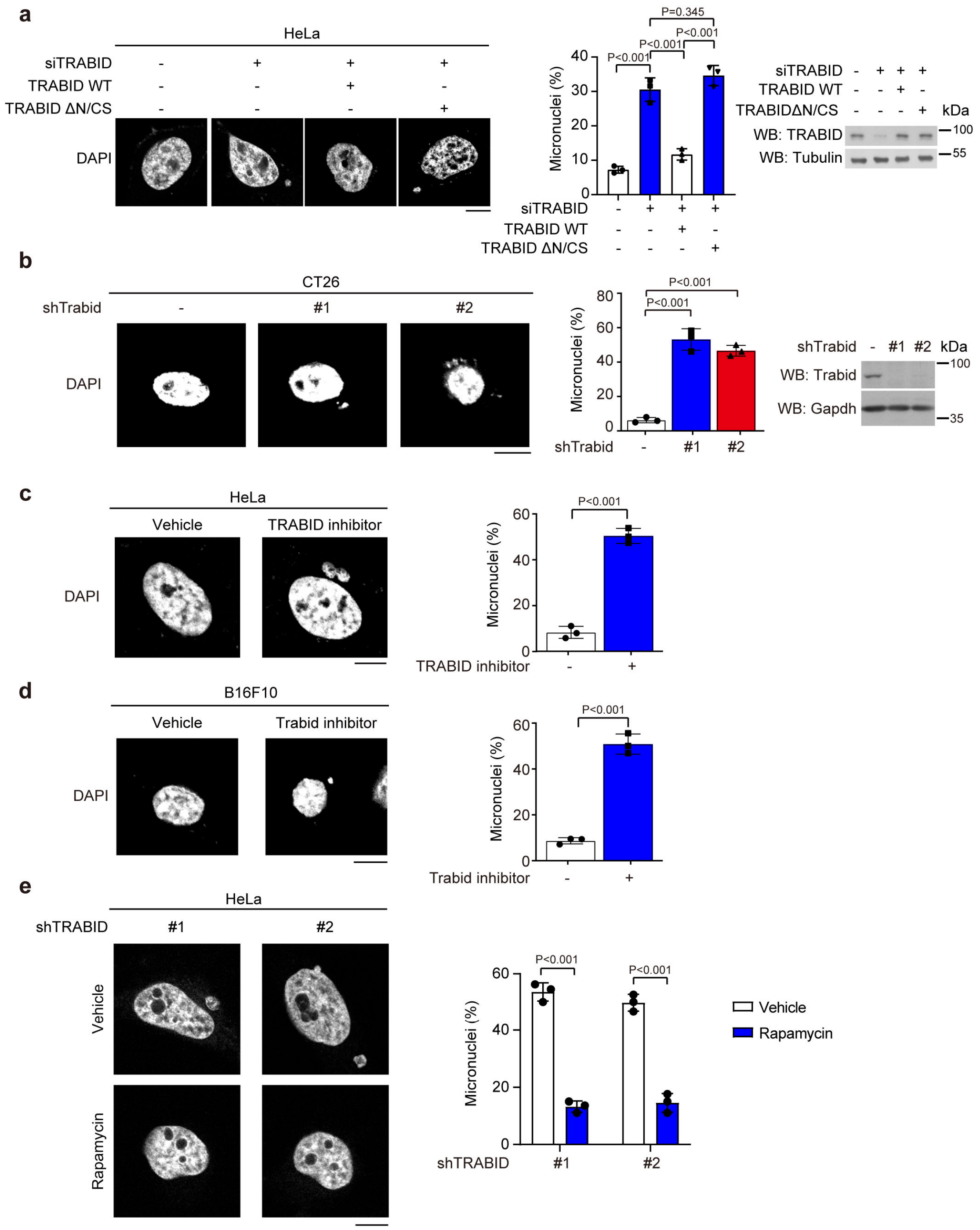

Supplementary Fig. 4. TRABID deficiency induces micronuclei, which is reversed by autophagy activation. (a) HeLa cells stably expressing TRABID or TRABID△N/CS were transfected with TRABID siRNA and stained with DAPI. Representative images, the percentages of cells with micronuclei, and the expression of TRABID are shown. Bar, 10 μm. Data are mean ± SD, (n=3 independent experiments and > 20 cells per group per experiment were counted). P values are

determined by two-way ANOVA with Tukey's post hoc test. (b-d) CT26 cells stably expressing control or TRABID shRNAs (b), or HeLa (c) or B16F10 (d) cells treated with 3 μM TRABID inhibitor for 24 h were stained with DAPI. Representative images and the percentages of cells with micronuclei are shown. Bars, 10 μm. Data are mean ± SD (n=3 independent experiments and > 20 cells per group per experiment were counted). P values are determined by one-way ANOVA with Tukey's post hoc test (b), or two-side Student's t-test (c, d). Trabid knockdown efficiencies are shown in b. (e) HeLa cells stably expressing TRABID shRNAs were treated with 100 nM Rapamycin for 24 h and then stained with DAPI. Representative images and the percentages of cells with micronuclei are shown. Bar, 10 μm. Data are mean ± SD (n=3 independent experiments and > 20 cells per group per experiment were counted). P values are determined by two-side Student's t-test. Blots are representatives of three (for b) or two (for a) independent experiments. Source data are provided as a Source Data file.

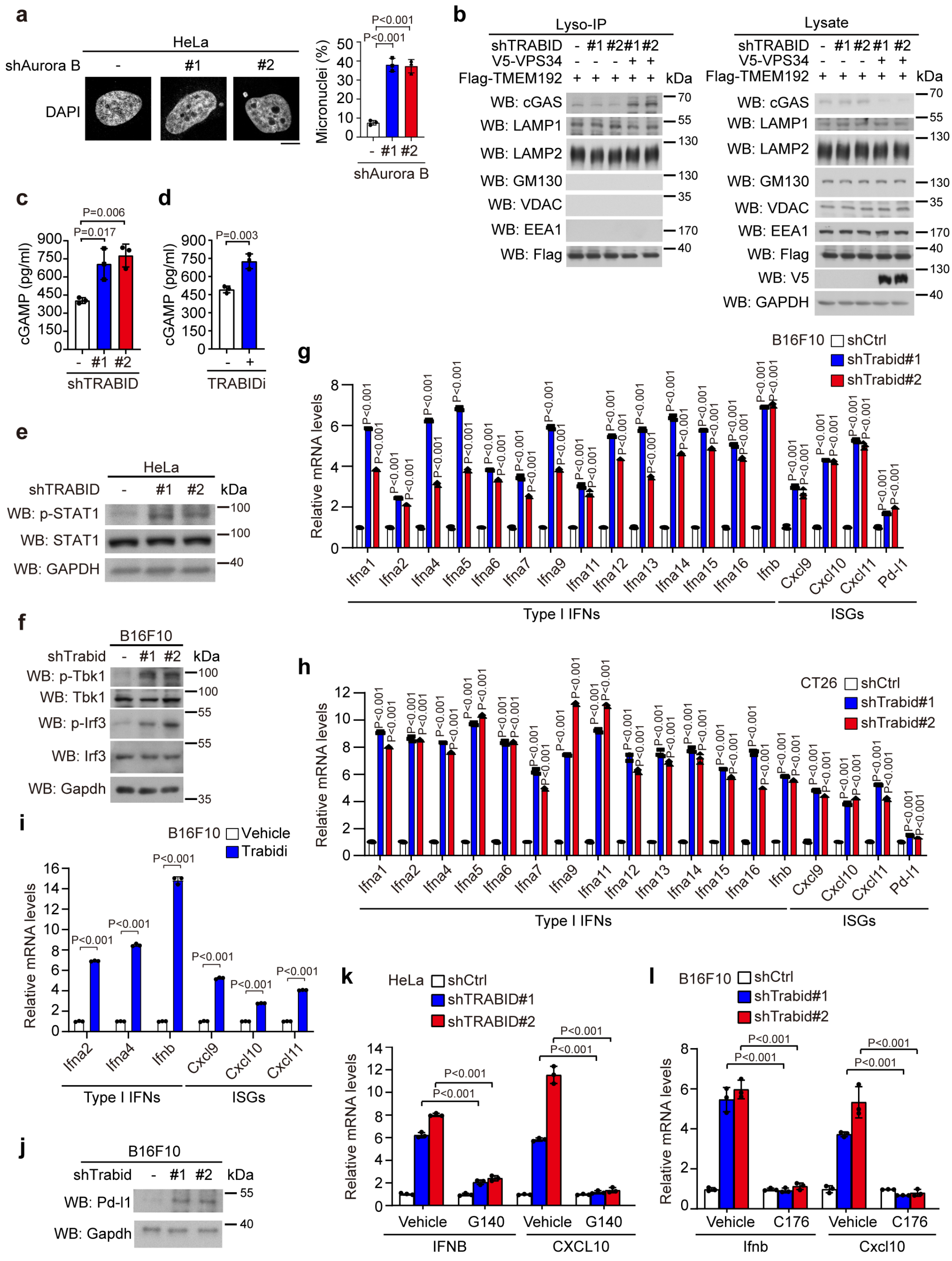

Supplementary Fig. 5. TRABID deficiency induces cGAS/STING pathway to turn on type I interferon responses. (a) HeLa cells stably expressing Aurora B shRNAs as in Supplementary Fig. 3g were stained with DAPI. Representative images and the percentages of cells with micronuclei are shown. Bar, 10 μm. Data are mean ± SD (n=3 independent experiments and > 20 cells per group per experiment were

counted). P values are determined by one-way ANOVA with Tukey's post hoc test. (b) Lyso-IP analysis of cGAS levels in lysosomes isolated from HeLa cells stably expressing TRABID shRNAs and transfected with Flag-TMEM192 and VPS34. (c, d) ELISA assay for cGAMP levels in HeLa cells stably expressing control or TRABID shRNAs (c), or HeLa cells treated with vehicle or 3 μM TRABID inhibitor for 24 h (d). (e, f, j) Western blot analysis of indicated proteins in HeLa cells (e) or B16F10 cells (f, j) stably expressing control or Trabid shRNAs. (g-i) RT-qPCR analysis of the expression of indicated genes in B16F10 (g) or CT26 (h) cells stably expressing control or Trabid shRNAs, or B16F10 cells treated with 3 μM TRABID inhibitor for 24 h (i). Data are normalized to control cells or untreated cells and expressed as fold changes. (k, l) RT-qPCR analysis of the expression of indicated genes in HeLa cells (k) or B16F10 cells (l) stably expressing control or TRABID shRNAs and treated with 6 μM G140 for 6 h (k) or 5 μM C176 for 16 h. Data are normalized to control cells and expressed as fold changes. Data in (c), (d), (g), (h), (i), (k), (l) are mean ± SD, n=3 independent experiments. P values are determined by one-way ANOVA with Tukey's post hoc test (c, g, h, k, l) or two-side Student's t-test (d, i). Blots are representatives of three (for f) or two (for b, e, j) independent experiments. Source data are provided as a Source Data file.

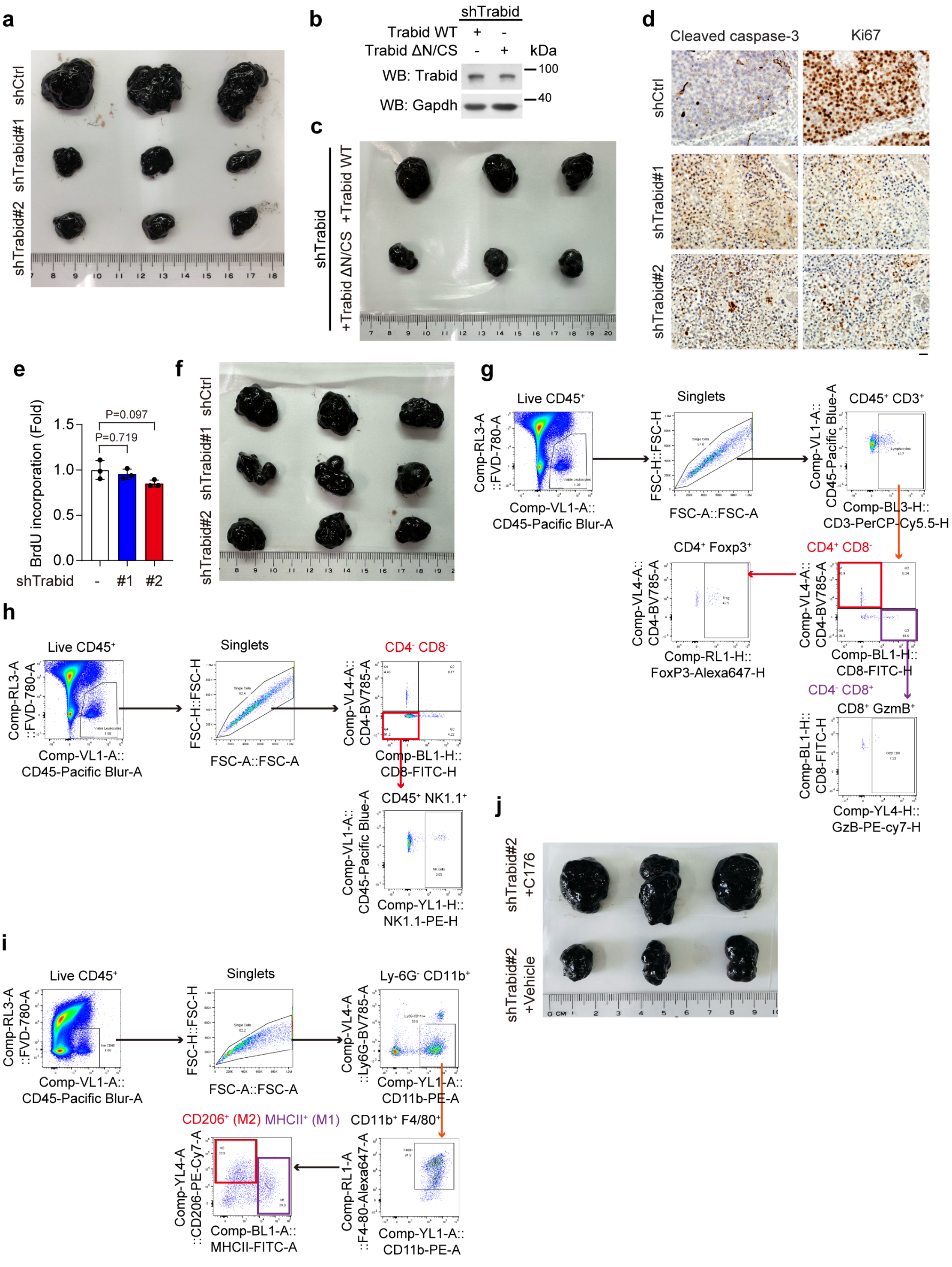

Supplementary Fig. 6. TRABID deficiency suppresses tumor growth through a TME- and STING-dependent mechanism. (a, c, f, j) Tumors surgically removed at day 15 or 18 in experiments shown in Fig. 6a, d, f, p are shown, respectively. (b) Western blot analysis of Trabid expression in B16F10 cells expressing Trabid shRNAs and reconstituted with Trabid wild type or ΔN/CS mutant. (d) IHC analyses

with indicated antibodies of the tumor sections derived from a. Bar, 20 μm. (e) BrdU analysis of the proliferation of B16F10 cells expressing control or TRABID shRNAs. Data are mean ± SD, n=3 independent experiments. P values are determined by one-way ANOVA with Tukey's post hoc test. (g-i) Gating strategies for indicated tumor-infiltrating immune cells. For (b) and (d), blots or images are representatives of two independent experiments. Source data are provided as a Source Data file.

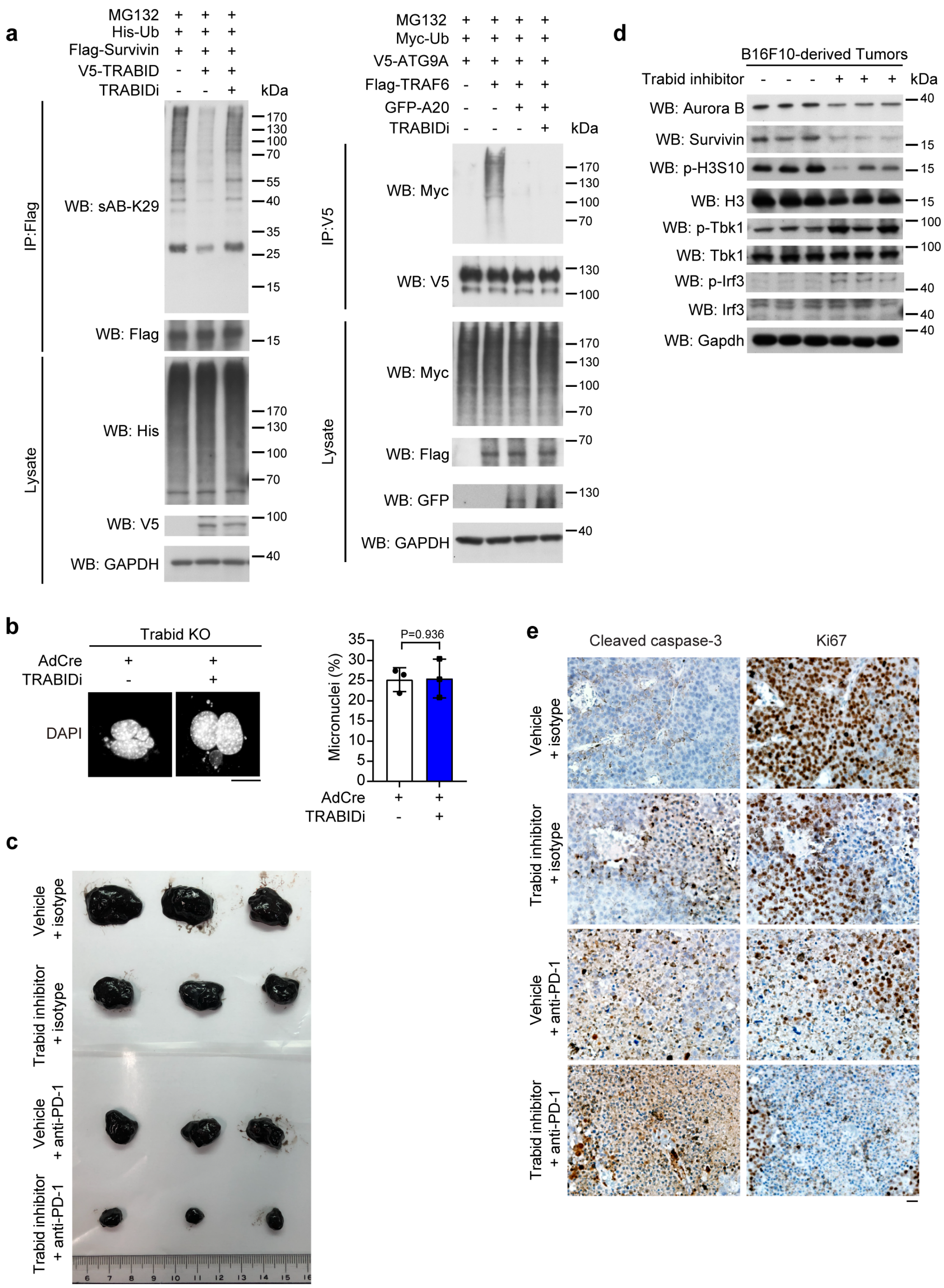

Supplementary Fig. 7. TRABID inhibition enhances the anti-tumor effects of anti-PD-1 antibody. (a) Analysis of Survivin or ATG9A ubiquitination in 293T cells transfected with indicated constructs and treated with 3 μM TRABID inhibitor for 16 h. Cells were treated with MG132 to preserve protein ubiquitination. (b) DAPI

staining of *Zranb1*$^{flox/flox}$ MEFs infected with AdCre (for Trabid KO) and simultaneously treated with or without 3 μM TRABID inhibitor for 48 h. Bar, 20 μm. The percentage of cells showing micronuclei was counted. Data are mean ± SD (n=3 independent experiments and > 200 cells per group per experiment were counted). P values are determined by two-side Student's t-test. (c) Tumors surgically removed at day 15 in experiment shown in Fig. 7a. (d, e) Western blot (d) and IHC (e) analyses for the expression of various proteins in tumors derived from a. Bar, 20 μm. Blots or images are representatives of three (for a) or two (for d, e) independent experiments. Source data are provided as a Source Data file.

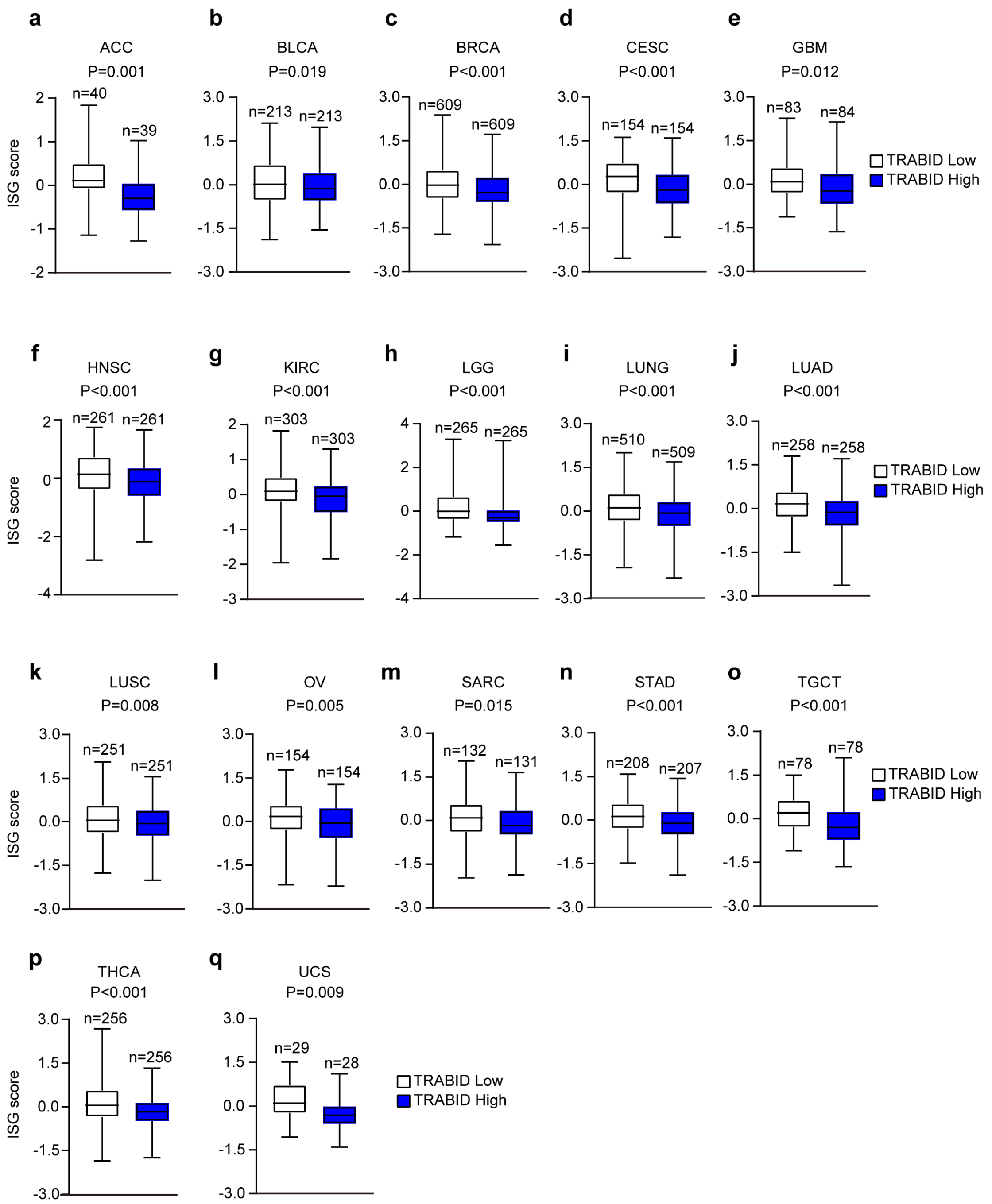

Supplementary Fig. 8. TRABID expression in various human cancer types correlates negatively with an interferon signature. (a-q) ISG scores in TRABID high and low expression groups of indicated TCGA cancer types. The patients are equally divided into two groups with indicated n numbers. Data are presented by Whiskers boxplot. The center line in the box indicates the median, the box represents the first and third quartiles and the whiskers represent the minimum and maximum. P

values are determined by two-side Student's t-test. Source data are provided as a Source Data file.

Supplementary Table 1: Antibody information

Protein	Vendor	Cat. number	Source	Clone number	Titer
Tubulin	GeneTex	GTX112141	Rabbit	N/A	WB (1:3000)
GAPDH	GeneTex	GTX100118	Rabbit	N/A	WB (1:5000)
Flag	GeneTex	GTX115043	Rabbit	N/A	WB (1:5000)
Histone H3	GeneTex	GTX122148	Rabbit	N/A	WB (1:1000)
p-Histone H3	GeneTex	GTX128116	Rabbit	N/A	WB (1:2000)
p-TBK1	GeneTex	GTX02815	Rabbit	N/A	WB (1:500)
p-IRF3	GeneTex	GTX02883	Rabbit	N/A	WB (1:500)
Survivin	GeneTex	GTX100052	Rabbit	N/A	WB (1:100)
INCENP	ABclonal	A0622	Rabbit	N/A	WB (1:1000)
Borealin	ABclonal	A15463	Rabbit	N/A	WB (1:1000)
Aurora B	ABclonal	A19539	Rabbit	ARC50905	WB (1:1000) PLA (1:250)
Aurora B	Abcam	Ab287960	Rabbit	N/A	IP (1:100)
Cyclin E1	BOSTER	M00543	Rabbit	GHB-3	WB (1:500)
Cyclin B1	Santa Cruz	Sc-245	Mouse	GNS1	WB (1:3000)
GFP	Santa Cruz	Sc-9996	Mouse	B-2	WB (1:500)
LC3	Abcam	Ab48394	Rabbit	N/A	IF (1:100)
6 x His	Takara Bio	631212	Mouse	unknown	WB (1:5000)
V5	Merck Millipore	AB3792	Rabbit	N/A	WB (1:3000) IF (1:5000)
V5	Invitrogen	R960-25	Mouse	SV5-Pk1	PLA (1:500)
p-STING	Cell Signaling	19781	Rabbit	N/A	WB (1:500)
TBK1	Cell Signaling	3504	Rabbit	N/A	WB (1:1000)
IRF3	Cell Signaling	11904	Rabbit	N/A	WB (1:1000)
IKKβ	Cell Signaling	2684	Rabbit	N/A	WB (1:1000)
p-IKKβ	Cell Signaling	2697	Rabbit	N/A	WB (1:500)
IκBα	Cell Signaling	9242	Rabbit	N/A	WB (1:1000)
p65	Cell Signaling	8242	Rabbit	N/A	WB (1:1000) IF (1:100)
p-p65	Cell Signaling	3033	Rabbit	N/A	WB (1:500)
LAMP1	Abcam	Ab25630	Mouse	H4A3	WB (1:1000)
LAMP2	Santa Cruz	Sc-18822	Mouse	H4B4	WB (1:1000)

GM130	Abcam	Ab32337	Rabbit	N/A	WB (1:1000)
GM130	BD Transduction Laboratories	AB_398142	Mouse	35	WB (1:1000)
ATP5A	Abcam	Ab14748	Mouse	15H4C4	WB (1:1000)
EEA1	GeneTex	GTX109638	Rabbit	N/A	WB (1:1000)
VDAC	Cell Signaling	4661	Rabbit	N/A	WB (1:2000)
TRABID (Ank)	Jackson ImmunoResearch Laboratories	109-036-006	Rabbit	N/A	WB (1:1000)
Myc-Tag	Cell Signaling	2278	Rabbit	N/A	WB (1:1000)
PD-L1 (Human)	Cell Signaling	13684	Rabbit	N/A	WB (1:1000)
PD-L1 (Mouse)	Abclonal	A18103	Rabbit	ARC5110-01	WB (1:1000)
p-STAT1	Cell Signaling	9167	Rabbit	N/A	WB (1:1000)
STAT1	Cell Signaling	9172	Rabbit	N/A	WB (1:1000)
F(ab)'2 Fragment Affinity-Purified Secondary antibody	Jackson ImmunoResearch Laboratories	109-036-006		N/A	WB (1:5000)
Rabbit IgG HRP	GE Healthcare	NA934	Donkey	N/A	WB (1:5000)
Mouse IgG HRP	GE Healthcare	NA931	Sheep	N/A	WB (1:5000)
cGAS	Cell Signaling	83623	Rabbit	N/A	WB (1:1000)
VPS34	Echelon Biosciences	Z-R016	Rabbit	N/A	WB (1:1000)
α-tubulin	Sigma	T6199	Mouse	DM1A	IF (1:2000)
anti-Mouse IgG, Alexa Fluor 488	Thermo Fisher Scientific	A-11001	Goat	N/A	IF (1:100)
CD16/32	BioLegend	101302	Rabbit	93	Flow (0.5

					µg/test)
CD45 (Pacific Blue)	BioLegend	103126	Rabbit	30-F11	Flow (0.25 µg/test)
CD4 (Brilliant Violet 785)	BioLegend	100552	Rabbit	RM4-5	Flow (0.25 µg/test)
CD8 (FITC)	BioLegend	100706	Rabbit	53-6.7	Flow (1 µg/test)
CD3 (PerCP/Cyanine5.5)	BioLegend	100218	Rabbit	17A2	Flow (2 µg/test)
NK1.1 (PE)	BioLegend	108708	Mouse	PK136	Flow (1 µg/test)
Foxp3 (Alexa Fluor 647)	BioLegend	126408	Rabbit	MF-14	Flow (1.5 µg/test)
Granzyme B (PE/Cyanine7)	BioLegend	372214	Mouse	QA18A28	Flow (5µg /test)
CD11b (PE)	BioLegend	101208	Rabbit	M1/70	Flow (0.25 µg/test)
Ly-6G (Brilliant Violet 785)	BioLegend	127645	Rabbit	1A8	Flow (0.5 µg/test)
F4/80 (Alexa Fluor 647)	BioLegend	123122	Rabbit	BM8	Flow (0.5 µg/test)
I-A/I-E; MHC II (FITC)	BioLegend	107606	Rabbit	M5/114.15.2	Flow (0.25 µg/test)
CD206 (PE/Cyanine7)	BioLegend	141720	Rabbit	C068C2	Flow (0.25 µg/test)
PD-1	Bio X Cell	BE0273	Rabbit	29F.1A12	5 mg/kg
Isotype	Bio X Cell	BE0089	Rabbit	2A3	5 mg/kg

Control					
Anti – TRABID (ABS13)	Merck Millipore	32160702	Rabbit	N/A	IP:1:150
Cleaved caspase-3	Cell Signaling	9664	Rabbit	N/A	IHC(1:2000)
Ki67	Abcam	Ab16667	Rabbit	N/A	IHC (1:200)
Pericentrin	Abcam	Ab4448	Rabbit	N/A	IF (1:100)

Supplementary Table 2: Sequences of shRNAs and siRNAs

shRNA	Target sequence (5' to 3')	SOURCE (IDENTIFIER)
Luciferase shRNA	CTTCGAAATGTCCGTTCGGTT	National RNAi Core Facility, Academia Sinica, Taiwan
TRABID shRNA#1	GCAGTAGTGGTAATAGCCAAA	National RNAi Core Facility, Academia Sinica, Taiwan
TRABID shRNA#2	GCTGGAAAGATTGGGAATCAT	National RNAi Core Facility, Academia Sinica, Taiwan
Trabid shRNA#1 (for mouse cell lines)	GCCTGCATGACTGTTCACATT	National RNAi Core Facility, Academia Sinica, Taiwan
Trabid shRNA#2 (for mouse cell lines)	GTCTGGACAGTAGACTATATG	National RNAi Core Facility, Academia Sinica, Taiwan
Aurora B shRNA#1	GCATCACACAACGAGACCTAT	National RNAi Core Facility, Academia Sinica, Taiwan
Aurora B shRNA#2	CCTGCGTCTCTACAACTATTT	National RNAi Core Facility, Academia Sinica, Taiwan
Survivin shRNA#1	CCGCATCTCTACATTCAAGAA	National RNAi Core Facility, Academia Sinica, Taiwan
Survivin shRNA#2	CCTTTCTGTCAAGAAGCAGTT	National RNAi Core Facility, Academia Sinica, Taiwan
Pooled TRABID siRNAs	Not available by vendor	Horizon Discovery (Cat# L-009270-00-0010)

Supplementary Table 3: Sequences of qPCR primers

Gene name		Sequence (5’ to 3’)
TRABID	F	TGTACCCAGTGCTTATCCCAA
	R	TGCTGTGTCCTAGTGTTCAGTT
GAPDH	F	TGTTGCCATCAATGACCCCTT
	R	CTCCACGACGTACTCAGCG
IFNB	F	GCTTGGATTCCTACAAAGAAGCA
	R	ATAGATGGTCAATGCGGCGTC
IFNA1	F	AGAAGGCTCCAGCCATCTCTGT
	R	TGCTGGTAGAGTTCGGTGCAGA
IFNA2	F	TGGGCTGTGATCTGCCTCAAAC
	R	CAGCCTTTTGGAACTGGTTGCC
IFNA4	F	GTTCCAGAAGGCTCAAGCCATC
	R	TAGGAGGCTCTGTTCCCAAGCA
IFNA5	F	GCCTGAGTAACAGGAGGACTTTG
	R	TGAGCCTTCTGGAACTGGTTGC
IFNA6	F	AAGGCTGAAGCCATCTCTGTCC
	R	AGCCTCTCATCCCAAGCAACAG
IFNA7	F	GAAGACTCAAGCCATCTCTGTCC
	R	TAGGAGGCTCTGTTCCCAAGCA
IFNA8	F	GTTCCAGAAGGCTCAAGCCATC
	R	GAAGGGTCTCATCCAAAGCAGC
IFNA10	F	GTTCCAGAAGGCTCAAGCCATC
	R	TAGGAGGCTCTGTTCCCAAGCA
IFNA14	F	TCCTGCCTGAAGGACAGACATG
	R	GCTGCATCATCTCATGGAGGAC
IFNA16	F	ATCTGCCTCAGACTCACAGCCT
	R	CTGGTTGCCATCAAACACCTCC
IFNA17	F	GAAGACTCAAGCCATCTCTGTCC
	R	TAGGAGGCTCTGTTCCCAAGCA
IFNA21	F	GTTCCAGAAGGCTCAAGCCATC
	R	GAGGCTCTGTTCCCAAGTAGCA
CXCL9	F	CCAGTAGTGAGAAAGGGTCGC
	R	AGGGCTTGGGGCAAATTGTT
CXCL10	F	GTGGCATTCAAGGAGTACCTC
	R	TGATGGCCTTCGATTCTGGATT
CXCL11	F	GACGCTGTCTTTGCATAGGC

	R	GGATTTAGGCATCGTTGTCCTTT
PD-L1	F	TGCCGACTACAAGCGAATTACTG
	R	CTGCTTGTCCAGATGACTTCGG
Trabid	F	CCAGACTCTAGTGCAAGACCA
	R	GGTCCTACGCTGGGATAAGC
Gapdh	F	AGGTCGGTGTGAACGGATTTG
	R	TGTAGACCATGTAGTTGAGGTCA
Ifnb	F	TGGGTGGAATGAGACTATTGTTG
	R	CTCCCACGTCAATCTTTCCTC
Ifna1	F	GGATGTGACCTTCCTCAGACTC
	R	ACCTTCTCCTGCGGGAATCCAA
Ifna2	F	TACTCAGCAGACCTTGAACCT
	R	CAGTCTTGGCAGCAAGTTGAC
Ifna4	F	GCAATGACCTCCATCAGCAGCT
	R	GTGGAAGTATGTCCTCACAGCC
Ifna5	F	GGATGTGACCTTCCTCAGACTC
	R	CACCTTCTCCTGTGGGAATCCA
Ifna6	F	GCAATGACCTCCATCAGCAGCT
	R	GTGGAAGTATGTCCTCACAGCC
Ifna7	F	TCCTGCCTGAAGGACAGAAAGG
	R	GGTCAGCTCATGCAGAACACAG
Ifna9	F	AGATCCAGGAGGCTCAAGCCAT
	R	CCAGTGCAGAATGAGTCTAGGAG
Ifna11	F	CGCATCAAAGGACTCATCTGCTG
	R	CTGCTGCATCAGACAACCTTGC
Ifna12	F	AGGATGTGACCTGCCTCAGACT
	R	TGATCTGCTGGGCATCCACCTT
Ifna13	F	AGGATGTGACCTGCCTCAGACT
	R	CACCTTCTCCTGTGGGAATCCA
Ifna14	F	TGACCTCAACACTCAGCTCAA
	R	AGGTGCCTGTATCTCTACCTG
Ifna15	F	GCAATGACCTCCATCAGCAGCT
	R	GTGGAAGTATGTCCTCACAGCC
Ifna16	F	AGGATGTGACCTGCCTCAGACT
	R	AGGGTATCCACCTTCTCCTGGG
Cxcl9	F	GGAGTTCGAGGAACCCTAGTG
	R	GGGATTTGTAGTGGATCGTGC

Cxcl10	F	CCAAGTGCTGCCGTCATTTTC
	R	GGCTCGCAGGGATGATTTCAA
Cxcl11	F	TGTAATTTACCCGAGTAACGGC
	R	CACCTTTGTCGTTTATGAGCCTT
Pd-l1	F	TGCGGACTACAAGCGAATCACG
	R	CTCAGCTTCTGGATAACCCTCG
