# Supplementary material for: TRABID inhibition activates cGAS/STING-mediated anti-tumor immunity through mitosis and autophagy dysregulation
Source: Nat Commun. 2023 May 26;14:3050. doi: 10.1038/s41467-023-38784-z (PMC10220035; doi:10.1038/s41467-023-38784-z)
Supplement: Supplementary file 3 — Description of Additional Supplementary Files [file 41467_2023_38784_MOESM3_ESM.pdf]

Description of Additional Supplementary Files

Supplementary Movie 1. Mitotic cell division in a control MEF cell. *Zranb1*$^{flox/flox}$ MEF cells infected with AdLuc were transduced with lentivirus carrying H2B-mCherry to mark the chromosomes. This video shows normal mitotic cell division. The images were taken every 2 min and they are played back at 5 frames per second.

Supplementary Movie 2. Mitotic cell division in a Trabid KO MEF cell. *Zranb1*$^{flox/flox}$ MEF cells infected with AdCre were transduced with lentivirus carrying H2B-mCherry to mark the chromosomes. This video shows the presence of chromosome mis-segregation. The images were taken every 2 min and they are played back at 5 frames per second.

Supplementary Movie 3. Mitotic cell division in a Trabid KO MEF cell. *Zranb1*$^{flox/flox}$ MEF cells infected with AdCre were transduced with lentivirus carrying H2B-mCherry to mark the chromosomes. This video shows the presence of chromosome bridge. The images were taken every 2 min and they are played back at 5 frames per second.

Supplementary Movie 4. Mitotic cell division in a control HeLa cell. HeLa cells expressing control shRNA were transduced with lentivirus carrying H2B-mCherry to mark the chromosomes. This video shows normal mitotic cell division. The images were taken every 2 min and they are played back at 5 frames per second.

Supplementary Movie 5. Mitotic cell division in a TRABID knockdown HeLa cell. HeLa cells expressing TRABID shRNA were transduced with lentivirus carrying H2B-mCherry to mark the chromosomes. This video shows the presence of lagging chromosome. The images were taken every 2 min and they are played back at 5 frames per second.
