# Supplementary material for: TRABID inhibition activates cGAS/STING-mediated anti-tumor immunity through mitosis and autophagy dysregulation
Source: Nat Commun. 2023 May 26;14:3050. doi: 10.1038/s41467-023-38784-z (PMC10220035; doi:10.1038/s41467-023-38784-z)
Supplement: Supplementary file 9 — Reporting Summary [file 41467_2023_38784_MOESM9_ESM.pdf]

nature portfolio

Corresponding author(s): Ruey-Hwa Chen

Last updated by author(s): April 30, 2023

# Reporting Summary

Nature Portfolio wishes to improve the reproducibility of the work that we publish. This form provides structure for consistency and transparency in reporting. For further information on Nature Portfolio policies, see our Editorial Policies and the Editorial Policy Checklist.

## Statistics

For all statistical analyses, confirm that the following items are present in the figure legend, table legend, main text, or Methods section.

| n/a | Confirmed | |
|---|---|---|
| [ ] | [x] | The exact sample size (*n*) for each experimental group/condition, given as a discrete number and unit of measurement |
| [ ] | [x] | A statement on whether measurements were taken from distinct samples or whether the same sample was measured repeatedly |
| [ ] | [x] | The statistical test(s) used AND whether they are one- or two-sided<br>*Only common tests should be described solely by name; describe more complex techniques in the Methods section.* |
| [ ] | [x] | A description of all covariates tested |
| [ ] | [x] | A description of any assumptions or corrections, such as tests of normality and adjustment for multiple comparisons |
| [ ] | [x] | A full description of the statistical parameters including central tendency (e.g. means) or other basic estimates (e.g. regression coefficient) AND variation (e.g. standard deviation) or associated estimates of uncertainty (e.g. confidence intervals) |
| [ ] | [x] | For null hypothesis testing, the test statistic (e.g. *F*, *t*, *r*) with confidence intervals, effect sizes, degrees of freedom and *P* value noted<br>*Give P values as exact values whenever suitable.* |
| [x] | [ ] | For Bayesian analysis, information on the choice of priors and Markov chain Monte Carlo settings |
| [x] | [ ] | For hierarchical and complex designs, identification of the appropriate level for tests and full reporting of outcomes |
| [ ] | [x] | Estimates of effect sizes (e.g. Cohen's *d*, Pearson's *r*), indicating how they were calculated |

*Our web collection on statistics for biologists contains articles on many of the points above.*

## Software and code

Policy information about availability of computer code

| | |
|---|---|
| Data collection | Proteome Discoverer 2.4.1.15 (2.4SP1), ZEISS ZEN2 image software (ZEN 2.6 blue edition), OLYMPUS FV3000 FV31S-SW (v 2.40), Attune NxT software (v 4.2.0) |
| Data analysis | GrapPad Prism v.9, ImageJ 1.53c , MaxQuant software (Version 1.6.15.0), Mascot search engine (v.2.7.0; Matrix Science, Boston, MA, USA), FlowJo (v10.8.1 and v8) , Phyton Statistical package Scipy stats (v1.9.0), TIMER (2.0). |

For manuscripts utilizing custom algorithms or software that are central to the research but not yet described in published literature, software must be made available to editors and reviewers. We strongly encourage code deposition in a community repository (e.g. GitHub). See the Nature Portfolio guidelines for submitting code & software for further information.

## Data

Policy information about availability of data

All manuscripts must include a data availability statement. This statement should provide the following information, where applicable:

- Accession codes, unique identifiers, or web links for publicly available datasets
- A description of any restrictions on data availability
- For clinical datasets or third party data, please ensure that the statement adheres to our policy

The original MS data for comparing proteome changes by TRABID knockdown are deposited to the ProteomeXchange Consortium via PRIDE partner repository with the project accession number PXD035002 (https://www.ebi.ac.uk/pride/archive/projects/PXD035002). The analyses in Fig. 8a and Supplementary Fig. 8a-q were acquired using TCGA database from online web server UCSC Xena Functional Genomics Explorer (https://xenabrowser.net/), whereas data in Fig. 8b, c were

retrieved from TIMER2.0 web platform (http://timer.comp-genomics.org/). The remaining data are available within the article, supplementary information, and source data file. Source data are provided with this paper.

## Human research participants

Policy information about studies involving human research participants and Sex and Gender in Research.

| | |
|---|---|
| Reporting on sex and gender | N/A |
| Population characteristics | N/A |
| Recruitment | N/A |
| Ethics oversight | N/A |

Note that full information on the approval of the study protocol must also be provided in the manuscript.

# Field-specific reporting

Please select the one below that is the best fit for your research. If you are not sure, read the appropriate sections before making your selection.

☒ Life sciences ☐ Behavioural & social sciences ☐ Ecological, evolutionary & environmental sciences

For a reference copy of the document with all sections, see nature.com/documents/nr-reporting-summary-flat.pdf

# Life sciences study design

All studies must disclose on these points even when the disclosure is negative.

| | |
|---|---|
| Sample size | No statistical method was used to predetermine sample size. For cell-based experiments, n=3 was chosen based on previous publications in the field (e.g., Nature Communications 9: 688, 2018; Science Translational Medicine 12: eaaz5683, 2020). For animal experiments, n=4-6 per group was chosen based on previous publications in the field (e.g., Nature Communications 13:1700, 2022; Science Translational Medicine 12: eaaz5683, 2020). |
| Data exclusions | No samples or animals were excluded from the analyses. |
| Replication | All experiments were repeated at least two or three times and reproducible results were obtained. |
| Randomization | For in vitro experiments, cells were based on gain or loss of function experiments with appropriate controls. Cells were seeded identically at the oneset of the experiments and randomized into the various treatment groups prior to beginning of treatment protocols. For in vivo analyses, mice were randomly allocated into different treatment groups. |
| Blinding | The experiments were not blinded due to feasibility. Samples were processed through standard procedures. |

# Reporting for specific materials, systems and methods

We require information from authors about some types of materials, experimental systems and methods used in many studies. Here, indicate whether each material, system or method listed is relevant to your study. If you are not sure if a list item applies to your research, read the appropriate section before selecting a response.

## Materials & experimental systems

| n/a | Involved in the study | |
|---|---|---|
| ☐ | ☒ | Antibodies |
| ☐ | ☒ | Eukaryotic cell lines |
| ☒ | ☐ | Palaeontology and archaeology |
| ☐ | ☒ | Animals and other organisms |
| ☒ | ☐ | Clinical data |
| ☒ | ☐ | Dual use research of concern |

## Methods

| n/a | Involved in the study | |
|---|---|---|
| ☒ | ☐ | ChIP-seq |
| ☐ | ☒ | Flow cytometry |
| ☒ | ☐ | MRI-based neuroimaging |

## Antibodies

| | |
|---|---|
| Antibodies used | All antibodies used are listed in Supplementary Table 1 |

Validation

All commercial antibodies were used according to profiles of manufacturers and validated by the manufacturers: TRABID, Merck Millipore, ABS13: validated for human IP (HeLa cells 1:150); V5, Merck Millipore, AB3792: not validated, but recommended for human and mouse WB(1:3000); Phospho-Histone H3 (Ser10), GeneTex, GTX128116: validated for human WB (HeLa cells 1:2000) and mouse WB (4T1 cells); Phospho-TBK1, GeneTex, GTX02815: validated for human WB (HeLa cells 1:500); Phospho-IRF3 (Ser396), GeneTex,GTX02883: validated for human WB (HT-29 cells 1:500); Phospho-STING (Ser366), Cell Signaling, 19781: validated for human WB (THP-1 cells 1:500),; Phospho-IKKα/β (Ser176/180), Cell Signaling, 2697: validated for human WB (THP-1 cells 1:500); Phospho-NF-κB p65 (Ser536), Cell Signaling, 3033: validated for human WB (HeLa cells 1:500) and mouse WB (NIH/3T3 cells); alpha Tubulin, GeneTex, GTX112141: validated for human WB (293T, A431, HeLa, HepG2 cells 1:3000) and mouse WB (NIH/3T3 cells),; GAPDH, GeneTex, GTX100118: validated for human WB (293T, A431, HeLa, HepG2 cells 1:5000) and mouse WB (NIH/3T3 cells); DDDDK Tag, GeneTex, GTX115043: validated for human WB (293T cells 1:5000); Histone H3, GeneTex, GTX122148: validated for human WB (293T cells 1:1000); Survivin, GeneTex, GTX100052: validated for human WB (293T, HeLa cells 1:100); INCENP, ABclonal, A0622: validated for human WB (HeLa, 293T cells 1:1000); CDCA8, ABclonal, A15463: validated for human WB (293T, A-549 cells 1:1000); Aurora B, ABclonal, A19539: validated for human WB (MCF7, A-549 cells 1:1000) and human IF (C6 cells 1:250), clone number:ARC50905; CCNE1, BOSTER, M00543: validated for human WB (HeLa cells 1:500),clone number:GHB-3; cyclin B1, Santa Cruz, sc-245: validated for human WB (HeLa cells 1:3000) and mouse WB (NIH/3T3 cells),clone number:CNS1; GFP, Santa Cruz, sc-9996: validated for WB (COS cells transfected with GFP-tagged proteins 1:5000),clone number:B-2; LC3, ABcam, ab48394: validated for human IF (HeLa cells 1:100); 6x His, Takara Bio, 631212: not validated, but recommended for human WB (1:5000); V5 Epitope Tag, Merck Millipore, AB3792: not validated, but recommended for human and mouse WB (1:3000) and IF (1:5000); V5 Tag, Invitrogen, R960-25: not validated, but recommended for human and mouse PLA (1:500),clone number:SV5-Pk1; TBK1/NAK, Cell Signaling, 3504: validated for human WB (HCT-116 cells 1:1000); IRF-3, Cell Signaling, 11904: validated for human WB (HeLa cells 1:1000); IKKβ, Cell Signaling, 2684: validated for human and mouse WB (HeLa and NIH/3T3 cells 1:1000); IκBα, Cell Signaling, 9242: validated for human WB (HeLa cells 1:1000); NF-κB p65, Cell Signaling, 8242: validated for human WB (HeLa cells 1:1000); cGAS, Cell Signaling, 83623: validated for human WB (HCT-116 cells 1:1000); Pericentrin, ABcam, Ab4448: validated for human and rabbit IF (HeLa and NIH/3T3 cells 1:100); α-Tubulin, Sigma, T6199: validated for mouse embryo fiborblasts IF (1:2000),clone number:DM1A; PD-1, Bio X Cell, BE0273: not validated, but recommended for mouse in vivo (5mg/kg); Isotype Control, Bio X Cell, BE0089: not validated, but recommended for human and mouse in vivo (5mg/kg); Cleaved Caspase-3 (Asp175), Cell Signaling, 9664: validated for mouse IHC (mouse embryo 1:2000); Ki67, Abcam, ab16667: not validated, but recommended for human and mouse IHC (1:200); Alexa Fluor 488, Thermo Fisher Scientific, A-11001: validated for human and mouse FC/FACS and IF (1:100); CD16/32, BioLegend, 101302, validated for mouse (C57BL/6 splenocytes 0.5μg),clone number:93; CD45 (Pacific Blue™), BioLegend, 103126, validated for mouse (C57BL/6 splenocytes 0.25μg),clone number:30-F11; CD4 (Brilliant Violet 785™), BioLegend, 100552, validated for mouse (C57BL/6 splenocytes 0.25μg),clone number:RM4-5; CD8 (FITC), BioLegend, 100706, validated for mouse (C57BL/6 splenocytes 1μg),clone number:53-6.7; CD3 (PerCP/Cyanine5.5), BioLegend, 100218, validated for mouse (C57BL/6 splenocytes 2μg),clone number:17A2; NK1.1 (PE), BioLegend, 108708, validated for mouse (C57BL/6 splenocytes 1μg),clone number:PK136; FoxP3 (Alexa Fluor® 647), BioLegend, 126408, validated for mouse (C57BL/6 splenocytes 1.5μg),clone number:MF-14; Granzyme B (PE/Cyanine7), BioLegend, 372214, validated for human (peripheral blood mononuclear cells 5μg),clone number:QA18A28; CD11b (PE), BioLegend, 101208, validated for mouse (C57BL/6 bone marrow cells 0.25μg),clone number:M1/70; Ly-6G (Brilliant Violet 785™), BioLegend, 127645, validated for mouse (C57BL/6 bone marrow cells 0.5μg),clone number:1A8; F4/80 (Alexa Fluor® 647), BioLegend, 123122, validated for mouse (Balb/c peritoneal macrophages 0.5μg),clone number:BM8; I-A/I-E (FITC), BioLegend, 107606, validated for mouse (C57BL/6 splenocytes 0.25μg),clone number:M5/114.15.2; CD206 (PE/Cyanine7), BioLegend, 141720, validated for mouse (Balb/c peritoneal macrophages 0.25μg),clone number:C068C2; p-STAT1 ,Cell Signaling, 9167: validated for human WB (HeLa cells 1:1000); STAT1,Cell Signaling, 9172: validated for human WB (A549 cells 1:1000); F(ab)'2 Fragment Affinity-Purified Secondary antibody,Jackson ImmunoResearch Laboratories,109-036-006: not validated WB (1:5000); PD-L1 (Mouse ), Abclonal, A18103,validated for human WB (A549 cells 1:1000),clone number:ARC5110-01; PD-L1 (Human),Cell Signaling ,13684, validated for human WB (KARPAS-299 cell 1:1000); Myc-Tag ,Cell Signaling ,2278,validated for cells overexpressing (Myc-Bcl-2 1:1000); VDAC ,Cell Signaling ,4661,validated for human WB (MCF-7 cells 1:2000); EEA1, GeneTex, GTX109638, validated for human WB (293T cells 1:1000); ATP5A ,Abcam ,Ab14748,validated for human WB (HepG2 cells 1:1000),clone number:15H4C4; GM130 ,BD Transduction Laboratories™, AB_398142,validated for mouse WB (rat brain cells 1:1000),clone number:35; GM130 ,Abcam, Ab32337,validated for WB (Drosophila S2 cells 1:1000); LAMP1 ,Abcam ,Ab25630,validated for mouse WB (Mouse liver cells 1:1000),clone number:H4A3; LAMP2 ,Santa Cruz ,Sc-18822, validated for human WB (U-937cells 1:1000),clone number:H4B4; VPS34, Echelon Biosciences ,Z-R016,validated for human WB (293T cells 1:1000); Rabbit IgG HRP,GE Healthcare,NA934, not validated WB (1:5000); Mouse IgG HRP, GE Healthcare, NA931, not validated WB (1:5000)

# Eukaryotic cell lines

Policy information about cell lines and Sex and Gender in Research

Cell line source(s)

HeLa (CCL-2), 293T (CRL-3216) cells were obtained from the American Type Culture Collection (ATCC, Manassas, VA, USA), whereas 293FT cells (R70007) were obtained from Thermo Fisher. B16F10 (ATCC CRL-6475) and CT26 (ATCC CRL-2638) cells were provided by Che-Ming Jack Hu (Academia Sinica, Taipei, Taiwan), whereas Atg5 KO MEFs were obtained from Guang-Chao Chen (Academia Sinica, Taipei, Taiwan). MEFs were isolated from E12.5 Zranb1flox/flox mouse (EM07669, The European Mouse Mutant Archive).

Authentication

STR profiling

Mycoplasma contamination

All cell lines were tested as negative for mycoplasma contamination.

Commonly misidentified lines
(See ICLAC register)

Commonly misidentified cell lines HeLa and 293T were used. They have been verified.

## Animals and other research organisms

Policy information about studies involving animals; ARRIVE guidelines recommended for reporting animal research, and Sex and Gender in Research

| | |
|---|---|
| Laboratory animals | Eight- or ten-week-old male C57BL/6J mice or Nude mice were used in this study. Mice were housed in a specific pathogen-free animal facility under temperature 22 ± 2°C) and humidity-controlled (55 ± 5%) conditions with 12-hour light/12-hour dark circadian cycle and access to food and water. |
| Wild animals | Study did not include wild animals. |
| Reporting on sex | Sex was not considered in the study design. |
| Field-collected samples | Study did not involve sample collection from the field. |
| Ethics oversight | All animal studies were approved by Institutional Animal Care and Use Committee, Academia Sinica. |

Note that full information on the approval of the study protocol must also be provided in the manuscript.

## Flow Cytometry

### Plots

Confirm that:

- [x] The axis labels state the marker and fluorochrome used (e.g. CD4-FITC).
- [x] The axis scales are clearly visible. Include numbers along axes only for bottom left plot of group (a 'group' is an analysis of identical markers).
- [x] All plots are contour plots with outliers or pseudocolor plots.
- [x] A numerical value for number of cells or percentage (with statistics) is provided.

### Methodology

| | |
|---|---|
| Sample preparation | Tumors were minced and digested with type I collagenase. The dissociated tumor cells were filtered through a strainer and then incubated with ACK lysis buffer to remove red blood cells. Dead cells were excluded using Fixable Viability Stain 780 (BD). Cells were washed and blocked by an anti-CD16/32 antibody (BioLegend). Next, cells were stained with various antibodies to immune cell markers. For intracellular staining, cells were fixed and permeabilized using the Transcription Factor Buffer Set (BD). The detailed methodology is described in the “flow cytometry analysis” section of Methods.<br>For cell cycle profiling, cells were fixed and stained with DAPI. |
| Instrument | Thermo Fisher Scientific Attune NxT Flow Cytometer |
| Software | FlowJo (v10.8.1 for TILs; v8 for cell cycle profiling) |
| Cell population abundance | Sample purity (>90%) was confirmed by flow cytometry |
| Gating strategy | The CD45+ live cells were first gated and then singlet cells were gated according to the pattern of FSC-H vs FSC-A. Next, CD4+ TILs were gated as CD3+CD4+CD8-. CD8+ TILs were gated as CD3+CD8+CD4-. Active CD8+ T cells were gated as CD3+CD8 +CD4-GzB+. Treg TILs were gated as CD3+CD4+CD8-Foxp3+. NK TILs were gated as CD8-CD4-NK1.1+. M1 macrophages were gated as Ly-6G-CD11b+F4/80+MHCII+ M2 macrophages were gated as Ly-6G-CD11b+F4/80+CD206+ The detailed gating strategies are shown in Supplementary Fig. 6c-e. |

- [x] Tick this box to confirm that a figure exemplifying the gating strategy is provided in the Supplementary Information.
